# Why Is Aging Conserved and What Can We Do about It?

**DOI:** 10.1371/journal.pbio.1002131

**Published:** 2015-04-29

**Authors:** Jason N. Pitt, Matt Kaeberlein

**Affiliations:** Department of Pathology, University of Washington, Seattle, Washington, United States of America

## Abstract

The field of aging research has progressed rapidly over the past few decades. Genetic modulators of aging rate that are conserved over a broad evolutionary distance have now been identified. Several physiological and environmental interventions have also been shown to influence the rate of aging in organisms ranging from yeast to mammals. Here we briefly review these conserved pathways and interventions and highlight some key unsolved challenges that remain. Although the molecular mechanisms by which these modifiers of aging act are only partially understood, interventions to slow aging are nearing clinical application, and it is likely that we will begin to reap the benefits of aging research prior to solving all of the mysteries that the biology of aging has to offer.

## Abstract 2

Old age is the single greatest risk factor for the leading causes of death in the developed world. Advances in aging research promise to alleviate the diseases of aging by targeting aging itself.

## Introduction

Aging is something everyone can relate to. From grandparents, to parents, and ultimately our own bodies, we are intimately familiar with the declines in form and function that accompany old age. Yet, we don’t all appear to age at the same rate. Many individuals are healthy and active well into their 70s, 80s, or even 90s, while others will suffer from chronic disease and disability by the time they reach their 40s or 50s. Those of us that have companion animals also observe that different animal species or even subspecies, as in the case of dog breeds, age at profoundly different rates (Box 1). Defining the factors that influence individual rates of aging is a major focus of aging research.

**Box 1. Variation in Aging Rates across and within Species**

Despite many decades of study, the mechanisms that underlie the dramatic differences in life span across species still remain unknown. Even among the most common laboratory models used in aging research, there is a large range of aging rates: *Caenorhabditis elegans* grow old and die within a matter of 3 weeks, while mice and rats take about 50 times as long to age. Humans, of course, can live on average about 80 years in developed countries, with a documented maximum life span of 122 years [1]. It is worth noting that these differences in species life span dwarf the magnitude of effects currently achievable from longevity-promoting interventions in the lab, which generally increase life span by 30%–50%.

In an effort to understand the mechanisms underlying species differences in aging rate, efforts have been made to identify phenotypes that correlate with interspecies longevity, and among the strongest is body size: larger animals tend to live longer than smaller animals when comparing across species [2]. The molecular mechanisms underlying the correlation between body size and longevity across species remain unknown, although evolutionary arguments can be invoked to explain this relationship. For example, smaller species tend to be prone to higher rates of predation in the wild, and, thus, there is selective pressure to reproduce early in life and, perhaps, age quickly. Metabolic rate has also been suggested to underlie this relationship, as large animals tend to have a lower metabolic rate when normalized for body size. This is somewhat controversial, however, as the methods used to measure and normalize for metabolic rate are not widely accepted or agreed upon.

Not surprisingly, there are numerous outliers in such correlational analyses, and efforts are being made to attempt to understand interspecies aging rates by studying these outliers. One well-known example is the naked mole rate, which is similar in size and closely related evolutionarily to other rodents but lives about ten times as long and may never get cancer [3]. Another example is certain species of clams that have exceptional longevity exceeding 300 years [4]. Other species of particular interest include those possibly exhibiting negligible senescence, such as the hydra, some plants, and certain species of turtles and rockfish [5].

Interestingly, the relationship between body size and longevity becomes inverted when comparing individuals within the same species: smaller individuals tend, on average, to live longer. Domestic dogs offer a particularly good example of this: small breeds such as the Chihuahua tend to live about twice as long, on average, as larger breeds such as the Great Dane. For dogs, one of the largest predictors of body size is the level of insulin-like growth factor 1 (IGF-1) activity [6], which has also emerged as a conserved longevity pathway in laboratory models (see Table 1). Although less pronounced in people than in dogs, the relationship between smaller body size and increased life expectancy also exists in humans, and one recent study suggests that rates of age-related diseases including cancer and diabetes are lower in humans with deficiency in growth hormone signaling [7].

**Table 1:** Some conserved pathways and interventions of aging.

Interventions	Yeast	Worms	Flies	Mice	Humans
**Environmental**					
Dietary Restriction	✓	✓	✓	✓	**
Lower Temperature	✓	✓	✓	✓	?
Low Oxygen	?	✓	✓	?	?
**Genetic**					
Insulin/ILS	✓	✓	✓	✓	**
mTOR/Rapamycin	✓	✓	✓	✓	**
AMPK/Metformin	✓	✓	✓	✓	**
Sirtuins/NAD	✓	✓	✓	✓	**
SOD/Catalase	✓	✓	✓	✓	?

**There is indirect evidence that each of these is associated with health span or longevity in humans. For specific examples, see references [8–49] and S1 Text. Abbreviations: AMPK, 5ʹ adenosine monophosphate-activated protein kinase; ILS, insulin-like signaling; mTOR, mechanistic target of rapamycin; NAD, nicotinamide adenine dinucleotide; SOD, superoxide dismutase.

**Fig 1:**
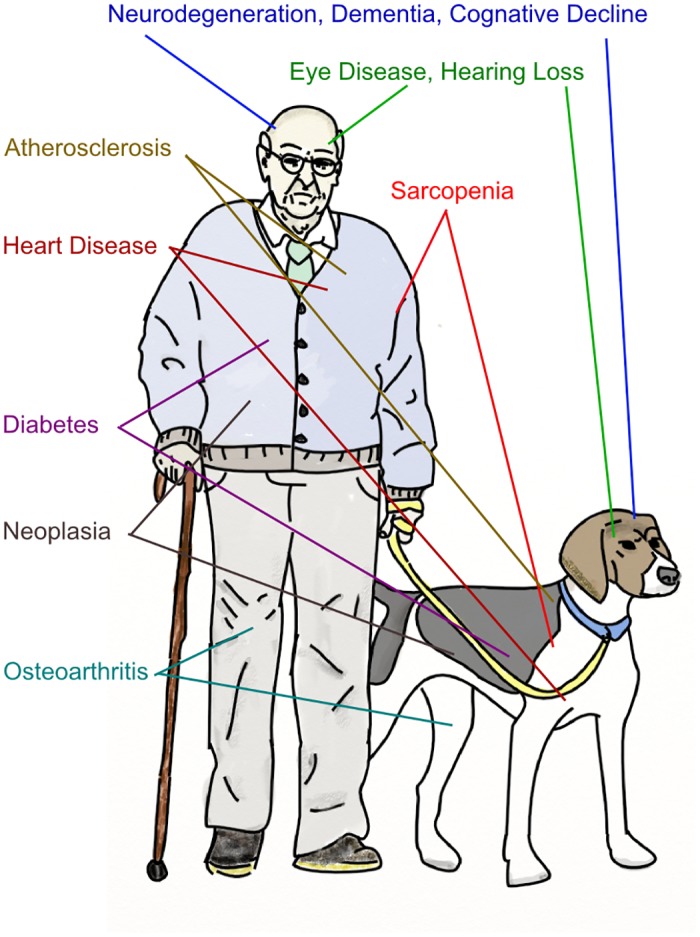
Common pathologies of aging. Incidence of disparate pathological conditions all showing a strong correlation with advanced human age. Many of these same conditions are also seen in aged dogs.

From a biomedical perspective, it is critically important to gain a better understanding of the mechanisms that drive biological aging, as age is the single greatest risk factor for the leading causes of death in developed nations [50]. The fact that aging influences so many different conditions is particularly curious (Fig 1). What is it about aging that creates an environment within our cells, tissues, and organs that is permissive for all of these seemingly disparate pathological states?

In order to understand the biological mechanisms of aging, scientists have turned to laboratory model organisms such as rats and mice, fruit flies, nematodes, and even yeast. While some have questioned the utility of these systems as models for human aging, it is now clear that similar pathways and processes affect longevity in each of these species. These studies have resulted in the identification of interventions that slow aging in taxa spanning broad evolutionary distances. Although it is still unknown whether these interventions will slow human aging, the potential impact on human health, if they do, is enormous.

## Evidence That Aging Mechanisms Are Conserved and Some Potential Explanations

There can no longer be any reasonable debate over whether shared environmental and genetic modifiers of longevity exist; several have been identified, and more will almost certainly be found (Table 1). This has been best studied in the context of dietary restriction (DR, also often referred to as caloric or calorie restriction, CR), which refers to a reduction in nutrient availability in the absence of malnutrition. A variety of different DR interventions have been shown to extend life span in a diverse array of organisms, including numerous studies in yeast, nematodes, fruit flies, mice, and rats, and one study in rhesus monkeys [51,52]. These observations have been strengthened by the discovery that genetic or pharmacological manipulation of key nutrient response pathways can have similar effects on longevity. Most notably, the insulin-like signaling (ILS)/ mechanistic target of rapamycin (mTOR) network has been found to play a central role in controlling life span in yeast, nematodes, fruit flies, and mice [53,54]. ILS and mTOR are both inhibited in response to DR and are thought to mediate many of the beneficial effects of DR on health and longevity. What remains less certain is whether these longevity factors, and the pathways they act within, are truly modulating the rate of biological aging and, if so, whether they do so by similar or divergent mechanisms in different organisms.

**Fig 2:**
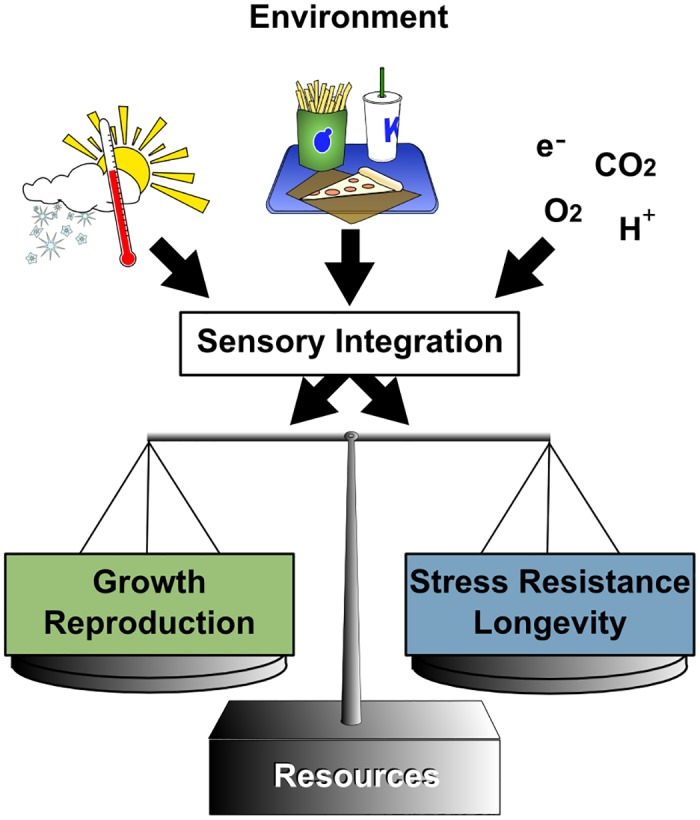
Environmental signals that modulate growth and reproduction also modulate aging. Organisms have evolved to grow and reproduce rapidly when environmental conditions, including temperature, oxygen levels, and food availability, are within optimal ranges. When these parameters are suboptimal but not lethal, organisms tend to become stress resistant and, perhaps as a by-product, longer lived. Conserved sensory pathways mediate this relationship, although there is also evidence that signals from the germ line can affect stress resistance and longevity and vice versa.

In general, the known conserved modifiers of longevity tend to mediate the relationship between fundamental environmental and physiological cues (i.e., temperature, nutrient status, and oxygen availability) and the regulation of growth and reproduction. One school of thought holds that this relationship results from the ability of organisms to forgo reproduction and invest in somatic maintenance during times of adversity [55]. In other words, based on the quality of the environment, the organism has evolved to make the appropriate choice between allocating its limited resources toward reproducing rapidly, and hence aging more quickly, versus delaying reproduction and allocating resources toward maintaining the soma, thereby aging more slowly. Lack of sufficient nutrients or other forms of environmental stress would thus tend to favor reduced signaling through growth promoting pathways, delayed reproduction, and longer life span. The idea that there is a direct trade-off between reproduction itself and longevity has been weakened by examples of long-lived mutants in both *C*. *elegans* and *Drosophila* that uncouple fecundity from life span extension [56,57]. At the same time, there is growing evidence that the sensing of the environmental cues is perhaps as important as the environmental composition itself [58]. This suggests that conserved sensory mechanisms coordinate both reproduction and longevity but that reproduction and longevity are distinct and that it is the investment in somatic maintenance, in response to stress, that results in greater longevity (Fig 2).

An alternative, but not mutually exclusive, model is that aging results from the continued expression of key developmental processes essential for growth and reproduction [59]. This “hyperfunction model” posits that although such growth-promoting pathways are beneficial—indeed necessary—early in life, their unregulated continuous activity becomes detrimental late in life and leads to many of the pathological conditions associated with old age [60]. Although the hyperfunction model has also been referred to as quasi-programmed aging, it is important to understand that, unlike programmed aging, this model does not postulate that natural selection has evolved a genetically encoded aging program [61]. True programmed aging, referring to an evolutionarily selected program of senescence, appears to be essentially nonexistent in animals [62]. Instead, longevity pathways are likely to have been selected to optimally regulate development, growth, and reproduction, with the resulting effects on aging a secondary consequence that is largely invisible to natural selection.

Based on this, we can speculate that conserved modifiers of aging exist because the sensory and signaling pathways that coordinate development, growth, reproduction, and stress resistance in response to environmental cues are themselves highly conserved. Whether the downstream mechanisms by which they affect life span are similarly conserved across species remains an open question that is discussed in greater detail below.

## What Are the Conserved Mechanisms of Aging?

Although conserved longevity pathways clearly exist, it has been challenging to identify their primary molecular mechanisms of action or even to definitively determine whether they directly modulate the rate of aging. This is true, in part, because there are no generally accepted molecular markers of aging rate in any organism (Box 2). In mammals, several phenotypes are known to correlate with chronological age, and a handful have been suggested to have some predictive power for future life expectancy; however, none have been demonstrated convincingly in prospective studies. One example is telomere length, which is often discussed as an “aging clock,” the idea being that our telomeres shorten as we get older and that this contributes to declines in tissue function. Despite the popularity of the concept, it remains unclear whether telomere shortening actually causes a majority of the pathological consequences of aging, and there is no consensus in the field on this question [63,64]. Similar arguments have been made for age-associated changes in hormonal levels, deregulation of gene expression, post-translational modification of circulating proteins, activation of transposable elements, changes in chromatin structure, stem cell exhaustion, and accumulation of somatic or mitochondrial DNA mutations [65,66], but to date none have been documented to provide quantitative measures of aging rate.

**Box 2. Biomarkers of Age and Aging Rate**

The identification of biomarkers of aging or biomarkers of aging rate has been a major goal of aging research for several decades. When considering biomarkers of aging, it is useful to differentiate between three important but different types of biomarkers: those that report on chronological age, those that report on biological age, and those that report on the rate of aging. Biomarkers of chronological age are intuitively straightforward: they provide quantitative measures of how old an individual is in units of time. Biomarkers of biological age, in contrast, provide quantitative measures of how old an individual is in units of the physiological progression from young to old. This is, unfortunately, difficult to define precisely, as we are still figuring out exactly what it means to be physiologically young and old. Biomarkers of aging rate provide quantitative measures of how quickly an organism is aging biologically. In other words, biological age can be considered as a function of chronological age multiplied by aging rate. The development of biomarkers of biological age or aging rate has the potential to greatly facilitate the development, validation, and translation of therapies to slow aging. In addition, such biomarkers could also be used to evaluate environmental or genetic factors that may accelerate the rate of aging, such as exposure to environmental toxins or unhealthy lifestyle choices.

From 1988–1998, the National Institute on Aging (NIA) sponsored a Biomarkers Initiative that included a series of workshops and a request for applications [66]. The results of this effort are now regarded as largely disappointing because the initiative resulted in no bona fide biomarkers of aging. This may be, in part, due to the fact that the technology was not available at the time to deeply probe molecular features of aging systems, and the only well-characterized intervention to slow aging at that time was dietary restriction. Advances since then have yielded numerous different ways to slow aging and extend longevity in model systems (see Table 1 for examples), and multiple studies have been aimed at identifying physiological, gene expression, proteomic, and metabolomic signatures that may be useful as biomarkers of aging rate [67].

One commonly discussed biomarker of biological age is telomere length. Telomeres shorten with age in most human tissues, and a prominent model of aging proposes that telomere shortening is the primary driver of age-associated cellular dysfunction and senescence. Shorter telomeres in blood cells have been associated with chronological age, disease, all-cause mortality, and environmental factors such as stress and poor diet; however, conflicting data from epidemiological studies and limitations to methodologies applied to measure telomere length have limited the utility of telomere length as a measure of biological age [68].

More recently, epigenetic changes to DNA in the form of methylation at specific sites have also emerged as a potential biomarker of aging. DNA methylation patterns appear to be predictive for chronological age across a variety of tissues, and one recent report claims that DNA methylation in blood can be used to predict all-cause mortality, even after controlling for other risk factors [69]. Additional studies will be needed to confirm this report; however, if such measures are sensitive enough, they may provide a useful approach to quantifying biological age and, perhaps, to testing the effects of interventions aimed at slowing aging rate.

If we define aging less specifically, to encompass the declines in cellular, organ, tissue and organismal function over time, then there is some evidence that the rate of aging can be slowed. For example, both DR and inhibition of mTOR with the drug rapamycin have been reported to delay the onset of multiple age-related phenotypes in mice, in addition to extending life span [52,70]. This includes reduced incidence of age-associated cancers, as well as improvements in age-related declines in cardiac, immune, kidney, liver, and cognitive function. In the case of DR, similar reductions in age-associated diseases have also been seen in rhesus monkeys [71]. However, not all phenotypes of aging are delayed in any of these cases, preventing a definitive answer to this question.

At a molecular level, conserved longevity pathways regulate numerous cellular processes that may contribute to their effects on health span and life span. In particular, a reduction in global mRNA translation, enhanced protein homeostasis, and improvements in mitochondrial function with age are associated with DR, reduced ILS, and inhibition of mTOR [50]. In mammals, these longevity-associated cellular changes are also associated with systemic changes, including a decrease in inflammatory processes that are likely to contribute to improved health [72,73]. The degree to which all of these changes, both individually and in concert, play a causal role in longevity and health span remains an area of active investigation, which may be informed by approaches that focus on conserved genomic, proteomic, and metabolomic features of aging.

## Toward a Solution

In addition to gaining an understanding of the molecular mechanisms of aging, a primary goal of aging research is to identify interventions that will slow aging in people. Advanced age is the primary risk factor for the majority of diseases in developed nations, and there are enormous social and financial pressures associated with demographic shifts toward more elderly populations. Interventions that expand the period of healthy life and reduce the period of chronic disease and disability (referred to as “compression of morbidity”) offer the potential to alleviate these pressures while simultaneously increasing individual productivity and quality of life [74,75].

In practical terms, it may not be necessary to understand in detail why aging is conserved in order to do something about it. In several cases, components of the insulin signaling/ mTOR network, as well as the sirtuins, have been shown to be associated with longevity and age-associated disease risk in people [33,76]. While it remains unclear how difficult it will be to develop interventions to improve healthy aging in humans, there is reason for optimism that this may not be far off. Drugs that target these pathways, including some already shown to increase life span and health span in rodents, are beginning to be tested for effects on age-associated phenotypes or disease in humans. Rapamycin, for example, has recently been shown to partially reverse age-associated declines in immune response to influenza vaccine in elderly humans [34], as had been seen previously in aged mice [77].

Unfortunately, because of the glacial pace of human aging when compared to common animal models, it will likely take several decades to determine whether rapamycin or other such compounds generally improve age-associated outcomes in people. We have recently proposed that companion animals, in particular mid- to large-size dogs, are an exceptionally good choice for bridging this gap through a midlife intervention trial with rapamycin (www.dogagingproject.com). Although there are some challenges, such as breed-specific susceptibilities to certain diseases, companion dogs offer many advantages for such a study: they share our environment, they suffer from many of the same age-related degenerative disorders that humans do and benefit from the same medical treatments [78], and it will be possible to obtain initial indications of efficacy as early as 6 months after the start of treatment (i.e., improvement in cardiac function), with survival outcomes available in a few years. Furthermore, the highly advanced state of veterinary medicine ensures that such a study can be done safely and that outcomes can be assessed accurately. Given that there are more than 70 million companion dogs in the United States alone, the impact of a successful intervention would be enormous and unprecedented, even if healthy life span is extended by only a year or two. Demonstrating the efficacy of rapamycin in dogs would not only greatly enhance the quality of life for companion animals and their owners but also provide invaluable information for trials seeking to promote healthy aging in people.

## Supporting Information

S1 Text Additional references and details of the experiments summarized in Table 1. (DOCX)

## Abbreviations

AMPK: 5' adenosine monophosphate-activated protein kinase
CR: caloric (or calorie) restriction
DR: dietary restriction
IGF-1: insulin-like growth factor 1
ILS: insulin-like signaling
mTOR: mechanistic target of rapamycin
NAD: nicotinamide adenine dinucleotide
NIA: National Institute on Aging
SOD: superoxide dismutase
