# Correction: Why Is Aging Conserved and What Can We Do about it?

**DOI:** 10.1371/journal.pbio.1002176

**Published:** 2015-05-15

**Authors:** Jason N. Pitt, Matt Kaeberlein

MK wishes to declare the following competing interest, which was omitted from the original article: MK is a founder of the Dog Aging Project, a University of Washington part-funded project that also solicits public donations through the University of Washington Foundation.
