# Supplementary material for: Why Is Aging Conserved and What Can We Do about It?
Source: PLoS Biol. 2015 Apr 29;13(4):e1002131. doi: 10.1371/journal.pbio.1002131 (PMC4414409; doi:10.1371/journal.pbio.1002131)
Supplement: S1 Text — (DOCX) [file pbio.1002131.s001.docx]

**Supplemental Information.**

**Expanded Table 1 Details.** Table 1 lists some interventions that have been reported to modulate aging in different organisms. For those readers who are interested in further reading, we provide additional references to the primary literature. This is by necessity not a comprehensive list, but provides key examples.

1. Dietary restriction has been shown to increase lifespan in yeast ([1](#_ENREF_1)), worms ([2](#_ENREF_2)), flies ([3](#_ENREF_3)) and mice ([4](#_ENREF_4)). There is some evidence that dietary restriction improves health and may slow age-related metabolic changes in humans ([5](#_ENREF_5)).
2. An inverse relationship between ambient temperature and lifespan has been observed in yeast ([6](#_ENREF_6)), worms ([7](#_ENREF_7)), and flies ([8](#_ENREF_8)). In mice, genetic alteration resulting in reduced core body temperature also leads to increased lifespan ([9](#_ENREF_9)).
3. Low oxygen (hypoxia) increases lifespan in worms ([10](#_ENREF_10)) and flies ([11](#_ENREF_11)).
4. Mutations that decrease insulin-like signaling increase lifespan in yeast ([12](#_ENREF_12)), worms ([13](#_ENREF_13)), flies ([14](#_ENREF_14)), and mice ([15](#_ENREF_15)). Mutations in the insulin-like growth factor 1 receptor ([16](#_ENREF_16)) and the FOXO3a transcription factor ([17](#_ENREF_17)), key components of this pathway, are associated with longevity in humans.
5. Inhibition of mTOR genetically or pharmacologically with rapamycin has been shown to increase lifespan in yeast ([18](#_ENREF_18),[19](#_ENREF_19)), worms ([20](#_ENREF_20),[21](#_ENREF_21)), flies ([22](#_ENREF_22),[23](#_ENREF_23)), and mice ([24](#_ENREF_24),[25](#_ENREF_25)). There is evidence that reduced expression of mTOR pathway genes is associated with longevity in people ([26](#_ENREF_26)) and that inhibition of mTOR with rapamycin may partially reverse age-associated declines in immune function in healthy individuals ([27](#_ENREF_27)).
6. Activation of AMPK genetically or pharmacologically through treatment with metformin increases lifespan in yeast ([28](#_ENREF_28)), worms ([29](#_ENREF_29),[30](#_ENREF_30)), flies ([31](#_ENREF_31)), and mice ([32](#_ENREF_32)). Diabetic patients taking metformin have reduced mortality relative to diabetic patients receiving sulphonylurea and potentially improved survival relative to non-diabetic controls ([33](#_ENREF_33)).
7. Overexpression of sirtuins in yeast ([34](#_ENREF_34)), worms ([35](#_ENREF_35)), flies ([36](#_ENREF_36)), and mice ([37](#_ENREF_37),[38](#_ENREF_38)) has been reported to extend lifespan. Polymorphisms in sirtuins have been associated with increased longevity in people ([39](#_ENREF_39)).
8. Overexpression or chemical mimetics of the antioxidant enzymes superoxide dismutase or catalase have been reported to extend lifespan in yeast ([12](#_ENREF_12)), worms ([40](#_ENREF_40)), flies ([41](#_ENREF_41)), and mice ([42](#_ENREF_42)).

**REFERENCES**

1. Lin, S. J., Defossez, P. A., and Guarente, L. (2000) Requirement of NAD and SIR2 for life-span extension by calorie restriction in Saccharomyces cerevisiae. *Science* **289**, 2126-2128.

2. Smith, E. D., Kaeberlein, T. L., Lydum, B. T., Sager, J., Welton, K. L., Kennedy, B. K., and Kaeberlein, M. (2008) Age- and calorie-independent life span extension from dietary restriction by bacterial deprivation in Caenorhabditis elegans. *BMC developmental biology* **8**, 49

3. Mair, W., Goymer, P., Pletcher, S. D., and Partridge, L. (2003) Demography of dietary restriction and death in Drosophila. *Science* **301**, 1731-1733

4. Weindruch, R., Walford, R. L., Fligiel, S., and Guthrie, D. (1986) The retardation of aging in mice by dietary restriction: longevity, cancer, immunity and lifetime energy intake. *The Journal of nutrition* **116**, 641-654

5. Holloszy, J. O., and Fontana, L. (2007) Caloric restriction in humans. *Exp Gerontol* **42**, 709-712

6. MacLean, M., Harris, N., and Piper, P. W. (2001) Chronological lifespan of stationary phase yeast cells; a model for investigating the factors that might influence the ageing of postmitotic tissues in higher organisms. *Yeast* **18**, 499-509

7. Leiser, S. F., Begun, A., and Kaeberlein, M. (2011) HIF-1 modulates longevity and healthspan in a temperature-dependent manner. *Aging Cell* **10**, 318-326

8. Sestini, E. A., Carlson, J. C., and Allsopp, R. (1991) The effects of ambient temperature on life span, lipid peroxidation, superoxide dismutase, and phospholipase A2 activity in Drosophila melanogaster. *Exp Gerontol* **26**, 385-395

9. Conti, B., Sanchez-Alavez, M., Winsky-Sommerer, R., Morale, M. C., Lucero, J., Brownell, S., Fabre, V., Huitron-Resendiz, S., Henriksen, S., Zorrilla, E. P., de Lecea, L., and Bartfai, T. (2006) Transgenic mice with a reduced core body temperature have an increased life span. *Science* **314**, 825-828

10. Leiser, S. F., Fletcher, M., Begun, A., and Kaeberlein, M. (2013) Life-span extension from hypoxia in Caenorhabditis elegans requires both HIF-1 and DAF-16 and is antagonized by SKN-1. *J Gerontol A Biol Sci Med Sci* **68**, 1135-1144

11. Rascon, B., and Harrison, J. F. (2010) Lifespan and oxidative stress show a non-linear response to atmospheric oxygen in Drosophila. *J Exp Biol* **213**, 3441-3448

12. Fabrizio, P., Pozza, F., Pletcher, S. D., Gendron, C. M., and Longo, V. D. (2001) Regulation of longevity and stress resistance by Sch9 in yeast. *Science* **292**, 288-290

13. Kenyon, C., Chang, J., Gensch, E., Rudner, A., and Tabtiang, R. (1993) A C. elegans mutant that lives twice as long as wild type. *Nature* **366**, 461-464

14. Clancy, D. J., Gems, D., Harshman, L. G., Oldham, S., Stocker, H., Hafen, E., Leevers, S. J., and Partridge, L. (2001) Extension of life-span by loss of CHICO, a Drosophila insulin receptor substrate protein. *Science* **292**, 104-106

15. Anisimov, V. N., and Bartke, A. (2013) The key role of growth hormone-insulin-IGF-1 signaling in aging and cancer. *Critical reviews in oncology/hematology* **87**, 201-223

16. Suh, Y., Atzmon, G., Cho, M. O., Hwang, D., Liu, B., Leahy, D. J., Barzilai, N., and Cohen, P. (2008) Functionally significant insulin-like growth factor I receptor mutations in centenarians. *Proc Natl Acad Sci U S A* **105**, 3438-3442

17. Willcox, B. J., Donlon, T. A., He, Q., Chen, R., Grove, J. S., Yano, K., Masaki, K. H., Willcox, D. C., Rodriguez, B., and Curb, J. D. (2008) FOXO3A genotype is strongly associated with human longevity. *Proc Natl Acad Sci U S A* **105**, 13987-13992

18. Kaeberlein, M., Powers, R. W., 3rd, Steffen, K. K., Westman, E. A., Hu, D., Dang, N., Kerr, E. O., Kirkland, K. T., Fields, S., and Kennedy, B. K. (2005) Regulation of yeast replicative life span by TOR and Sch9 in response to nutrients. *Science* **310**, 1193-1196

19. Powers, R. W., 3rd, Kaeberlein, M., Caldwell, S. D., Kennedy, B. K., and Fields, S. (2006) Extension of chronological life span in yeast by decreased TOR pathway signaling. *Genes Dev* **20**, 174-184

20. Vellai, T., Takacs-Vellai, K., Zhang, Y., Kovacs, A. L., Orosz, L., and Muller, F. (2003) Genetics: influence of TOR kinase on lifespan in C. elegans. *Nature* **426**, 620

21. Robida-Stubbs, S., Glover-Cutter, K., Lamming, D. W., Mizunuma, M., Narasimhan, S. D., Neumann-Haefelin, E., Sabatini, D. M., and Blackwell, T. K. (2012) TOR signaling and rapamycin influence longevity by regulating SKN-1/Nrf and DAF-16/FoxO. *Cell Metab* **15**, 713-724

22. Kapahi, P., Zid, B. M., Harper, T., Koslover, D., Sapin, V., and Benzer, S. (2004) Regulation of lifespan in Drosophila by modulation of genes in the TOR signaling pathway. *Curr Biol* **14**, 885-890

23. Bjedov, I., Toivonen, J. M., Kerr, F., Slack, C., Jacobson, J., Foley, A., and Partridge, L. (2010) Mechanisms of life span extension by rapamycin in the fruit fly Drosophila melanogaster. *Cell Metab* **11**, 35-46

24. Harrison, D. E., Strong, R., Sharp, Z. D., Nelson, J. F., Astle, C. M., Flurkey, K., Nadon, N. L., Wilkinson, J. E., Frenkel, K., Carter, C. S., Pahor, M., Javors, M. A., Fernandez, E., and Miller, R. A. (2009) Rapamycin fed late in life extends lifespan in genetically heterogeneous mice. *Nature* **460**, 392-395

25. Wu, J. J., Liu, J., Chen, E. B., Wang, J. J., Cao, L., Narayan, N., Fergusson, M. M., Rovira, II, Allen, M., Springer, D. A., Lago, C. U., Zhang, S., DuBois, W., Ward, T., deCabo, R., Gavrilova, O., Mock, B., and Finkel, T. (2013) Increased mammalian lifespan and a segmental and tissue-specific slowing of aging after genetic reduction of mTOR expression. *Cell reports* **4**, 913-920

26. Passtoors, W. M., Beekman, M., Deelen, J., van der Breggen, R., Maier, A. B., Guigas, B., Derhovanessian, E., van Heemst, D., de Craen, A. J., Gunn, D. A., Pawelec, G., and Slagboom, P. E. (2013) Gene expression analysis of mTOR pathway: association with human longevity. *Aging Cell* **12**, 24-31

27. Mannick, J. B., Del Giudice, G., Lattanzi, M., Valiante, N. M., Praestgaard, J., Huang, B., Lonetto, M. A., Maecker, H. T., Kovarik, J., Carson, S., Glass, D. J., and Klickstein, L. B. (2014) mTOR inhibition improves immune function in the elderly. *Sci Transl Med* **6**, 268ra179

28. Lu, J. Y., Lin, Y. Y., Sheu, J. C., Wu, J. T., Lee, F. J., Chen, Y., Lin, M. I., Chiang, F. T., Tai, T. Y., Berger, S. L., Zhao, Y., Tsai, K. S., Zhu, H., Chuang, L. M., and Boeke, J. D. (2011) Acetylation of yeast AMPK controls intrinsic aging independently of caloric restriction. *Cell* **146**, 969-979

29. Apfeld, J., O'Connor, G., McDonagh, T., DiStefano, P. S., and Curtis, R. (2004) The AMP-activated protein kinase AAK-2 links energy levels and insulin-like signals to lifespan in C. elegans. *Genes Dev* **18**, 3004-3009

30. Onken, B., and Driscoll, M. (2010) Metformin induces a dietary restriction-like state and the oxidative stress response to extend C. elegans Healthspan via AMPK, LKB1, and SKN-1. *PLoS One* **5**, e8758

31. Slack, C., Foley, A., and Partridge, L. (2012) Activation of AMPK by the putative dietary restriction mimetic metformin is insufficient to extend lifespan in Drosophila. *PLoS One* **7**, e47699

32. Martin-Montalvo, A., Mercken, E. M., Mitchell, S. J., Palacios, H. H., Mote, P. L., Scheibye-Knudsen, M., Gomes, A. P., Ward, T. M., Minor, R. K., Blouin, M. J., Schwab, M., Pollak, M., Zhang, Y., Yu, Y., Becker, K. G., Bohr, V. A., Ingram, D. K., Sinclair, D. A., Wolf, N. S., Spindler, S. R., Bernier, M., and de Cabo, R. (2013) Metformin improves healthspan and lifespan in mice. *Nature communications* **4**, 2192

33. Bannister, C. A., Holden, S. E., Jenkins-Jones, S., Morgan, C. L., Halcox, J. P., Schernthaner, G., Mukherjee, J., and Currie, C. J. (2014) Can people with type 2 diabetes live longer than those without? A comparison of mortality in people initiated with metformin or sulphonylurea monotherapy and matched, non-diabetic controls. *Diabetes, obesity & metabolism* **16**, 1165-1173

34. Kaeberlein, M., McVey, M., and Guarente, L. (1999) The SIR2/3/4 complex and SIR2 alone promote longevity in Saccharomyces cerevisiae by two different mechanisms. *Genes Dev* **13**, 2570-2580

35. Tissenbaum, H. A., and Guarente, L. (2001) Increased dosage of a sir-2 gene extends lifespan in Caenorhabditis elegans. *Nature* **410**, 227-230.

36. Rogina, B., and Helfand, S. L. (2004) Sir2 mediates longevity in the fly through a pathway related to calorie restriction. *Proc Natl Acad Sci U S A* **101**, 15998-16003

37. Kanfi, Y., Naiman, S., Amir, G., Peshti, V., Zinman, G., Nahum, L., Bar-Joseph, Z., and Cohen, H. Y. (2012) The sirtuin SIRT6 regulates lifespan in male mice. *Nature* **483**, 218-221

38. Satoh, A., Brace, C. S., Rensing, N., Cliften, P., Wozniak, D. F., Herzog, E. D., Yamada, K. A., and Imai, S. (2013) Sirt1 extends life span and delays aging in mice through the regulation of Nk2 homeobox 1 in the DMH and LH. *Cell Metab* **18**, 416-430

39. Bellizzi, D., Rose, G., Cavalcante, P., Covello, G., Dato, S., De Rango, F., Greco, V., Maggiolini, M., Feraco, E., Mari, V., Franceschi, C., Passarino, G., and De Benedictis, G. (2005) A novel VNTR enhancer within the SIRT3 gene, a human homologue of SIR2, is associated with survival at oldest ages. *Genomics* **85**, 258-263

40. Melov, S., Ravenscroft, J., Malik, S., Gill, M. S., Walker, D. W., Clayton, P. E., Wallace, D. C., Malfroy, B., Doctrow, S. R., and Lithgow, G. J. (2000) Extension of life-span with superoxide dismutase/catalase mimetics. *Science* **289**, 1567-1569

41. Orr, W. C., and Sohal, R. S. (1994) Extension of life-span by overexpression of superoxide dismutase and catalase in Drosophila melanogaster. *Science* **263**, 1128-1130.

42. Schriner, S. E., Linford, N. J., Martin, G. M., Treuting, P., Ogburn, C. E., Emond, M., Coskun, P. E., Ladiges, W., Wolf, N., Van Remmen, H., Wallace, D. C., and Rabinovitch, P. S. (2005) Extension of murine life span by overexpression of catalase targeted to mitochondria. *Science* **308**, 1909-1911
